# The challenge to avoid anti-malarial medicine stock-outs in an era of funding partners: the case of Tanzania

**DOI:** 10.1186/1475-2875-13-181

**Published:** 2014-05-11

**Authors:** Inez Mikkelsen-Lopez, Winna Shango, Jim Barrington, Rene Ziegler, Tom Smith, Don deSavigny

**Affiliations:** 1 Swiss Tropical and Public Health Institute, Basel, Switzerland; 2 University of Basel, Basel, Switzerland; 3 Ministry of Health and Social Welfare, Dar es Salaam, Tanzania; 4 Novartis Pharma AG, Basel, Switzerland

**Keywords:** Anti-malarial, Health systems, Tanzania, Accountability, Supply chain management, Stock-outs, Global fund

## Abstract

**Background:**

Between 2007 and 2013, the Tanzanian public sector received 93.1 million doses of first-line anti-malarial artemisinin-based combination therapy (ACT) in the form of artemether-lumefantrine entirely supplied by funding partners. The introduction of a health facility ACT stock monitoring system using SMS technology by the National Malaria Control Programme in mid 2011 revealed a high frequency of stock-outs of ACT in primary care public health facilities. The objective of this study was to determine the pattern of availability of ACT and possible causes of observed stock-outs across public health facilities in Tanzania since mid-2011.

**Methods:**

Data were collected weekly by the mobile phone reporting tool *SMS for Life* on ACT availability from over 5,000 public health facilities in Tanzania starting from September 2011 to December 2012. Stock data for all four age-dose levels of ACT across health facilities were summarized and supply of ACT at the national level was also documented.

**Results:**

Over the period of 15 months, on average 29% of health facilities in Tanzania were completely stocked out of all four-age dose levels of the first-line anti-malarial with a median duration of total stock-out of six weeks. Patterns of total stock-out by region ranged from a low of 9% to a high of 52%. The ACT stock-outs were most likely caused by: a) insufficient ACT supplies entering Tanzania (e.g. in 2012 Tanzania received 10.9 million ACT doses compared with a forecast demand of 14.4 million doses); and b) irregular pattern of ACT supply (several months with no ACT stock).

**Conclusion:**

The reduced ACT availability and irregular pattern of supply were due to cumbersome bureaucratic processes and delays both within the country and from the main donor, the Global Fund to Fight AIDS, Tuberculosis and Malaria. Tanzania should invest in strengthening both the supply system and the health information system using mHealth solutions such as *SMS for Life*. This will continue to assist in tracking ACT availability across the country where all partners work towards more streamlined, demand driven and accountable procurement and supply chain systems.

## Background

Malaria is of leading public health concern in Tanzania, especially for children under the age of five and pregnant women. According to the latest Tanzania HIV/AIDS and Malaria Indicator Survey in 2011–12, the prevalence of malaria rapid diagnostic test (mRDT) -confirmed malaria in children under five was 9% [1]. This is an important reduction compared to findings from the previous five years in the 2007–08 Malaria Indicator Survey, when 18% of children under five tested positive for malaria [2]. Expenditure on malaria interventions (including prevention and curative) accounts for 19.4% of total health expenditure and 1.6% of Gross Domestic Product in Tanzania [3], although the domestic budget for malaria activities has decreased by around 30% since 2005 [4]. The disease places a large burden on the country’s health sector, accounting for around 40% of outpatient department diagnoses in 2008 [5].

At the end of 2006, Tanzania changed its policy on first-line anti-malarial treatment for uncomplicated malaria to the use of artemisinin-based combination therapy (ACT) - artemether-lumefantrine (ALu) [2]; due to the high cost of ACT, Tanzania received USD 75 million from the Global Fund to Fight AIDS, Tuberculosis and Malaria (henceforth referred to as the Global Fund) during its Round 4 disbursements in 2005 (continuing currently through Round 9) to purchase ACT for use in the public sector. In rural Tanzania, for fever it has been shown that 58% of the population access their health services from public health facilities and the remaining 42% access treatment from the private sector, including faith-based organizations [6].

Tanzania’s Ministry of Health and Social Welfare (MoHSW), through its National Malaria Control Programme (NMCP), is responsible for forecasting ACT demand and managing Global Fund grants and the President’s Malaria Initiative (PMI) ACT supplies for the malaria programme [7]. The Pharmaceutical Services Section (PSS) is tasked with developing policy on pharmaceutical services and technologies. The Medical Stores Department (MSD), a parastatal of the MoHSW, is charged with handling health commodities for the public sector, including ACT procurement, storage and distribution [8]. Public health facilities order ACT quarterly along with other medicines through a “pull” system via the Integrated Logistic System (ILS) [9]. ACT are provided to the public from government health facilities and, according to policy, are dispensed free to children under the age of five years and to adults over 60 years [10]. Others pay a user fee in the public sector of TZS 1000 (USD 0.70) (fee since 2007) [11]. As stated in the 2005 Integrated Management of Childhood Illnesses Guidelines, ACT are given as a presumptive treatment in the absence of diagnostic tests when a child reports with fever without other symptoms, such as rapid breathing or other respiratory symptoms, which could indicate pneumonia or a common cold [12]. However recognizing that presumptive treatment may result in over prescribing of ACT, mRDTs were rolled out in Tanzania in early 2009, with national coverage by early 2012, for routine use in all levels of care for parasitological confirmation of malaria [13]. Apart from ACT, other anti-malarials offered include sulphadoxine–pyrimethamine (SP), which is recommended only as intermittent preventive treatment during pregnancy, and quinine, which is a second-line treatment for cases contra-indicated for ACT, administered to pregnant women in their first trimester, or in cases of severe malaria not responding to first-line treatment [14].

The design of the ACT procurement and delivery system is intended to guarantee routine availability in the public sector. Nonetheless, Tanzania, along with its neighbours, has been experiencing public sector ACT stock-outs over the past five years [7,15-21] with serious consequences for health care delivery and also for household health expenditure, as seen in the Ulanga District in Tanzania [6]. The objective of this study is to describe ACT availability across public health facilities in Tanzania and highlight some of the factors that influence it.

## Methods

Data on the availability of ACT were obtained from the *SMS for Life* reporting system [22], which was originally developed under a partnership consisting of the NMCP, Roll Back Malaria, Novartis Pharma AG, Vodafone, IBM, Medicines for Malaria Venture, and the Swiss Agency for Development and Cooperation, and which is now fully owned and operated by the MoHSW with support from the Global Fund. The *SMS for Life* system monitors weekly ACT stock levels via a mobile phone Short Message Service (SMS) for all four age-specific dose levels of ACT plus quinine. The ACT doses are divided into individual colour-coded blister packets according to weight of the patient: yellow packs for infants weighing 5 kg to under 15 kg; blue packs for toddlers weighing 15 kg to under 25 kg; red packs for children weighing 25 kg to under 35 kg; and, green packs for children weighing more than 35 kg and adults. Weekly SMS prompts are sent to designated primary care facility health workers in each health facility on their personal phones. Facility health workers are then required to report back within 27 hours on the stock count of full boxes of ACT in the store room for each of the four types. These messages are free of charge and if the health worker reports within 27 hours, they receive a credit on their phone (TZS 1,000, equivalent to USD 0.70) for personal use (pay for performance). Weekly summary and detailed status reports of stock situations in each facility are subsequently provided automatically to the District Medical Officer (DMO) and the District Pharmacist. This helps to determine and indicate which health facilities are at risk of stocking out, or actually stocked out of any dose level of ACT. The data are also made available on a password-protected website which displays current and historical stock status by health facility in a user-friendly, dashboard-driven application. Information is provided both graphically in trend format and on weekly-updated, interactive maps for greater interpretability. The website is available to the DMOs, the Regional Medical Officers, the NMCP, the MSD and the MoHSW-PSS and others stakeholders including Population Services International (PSI), Medicines for Malaria Venture (MMV) and the Swiss Agency for Development and Cooperation (SDC). The *SMS for Life* system began as a 21-week pilot in October 2009 covering 129 health facilities in three rural districts: Kigoma Rural (Kigoma Region), Lindi Rural (Lindi Region) and Ulanga (Morogoro Region). It was rolled out nationally two years later across all 132 districts, eventually covering 5,014 public health facilities by September 2011.

Data were collected from *SMS for Life* for 15 months, from October 2011 to December 2012 inclusive, for this study. The data, comprising the weekly numbers of boxes in stock by colour-code, for each facility, were downloaded in delimited ASCII format and tabulated using SAS v9.3 statistical software. A small number of facilities reported stocks that were multiples of thirty of plausible numbers of boxes. It was assumed these reports referred to numbers of blister-packs rather than of boxes, and divided accordingly.

**Figure 1:**
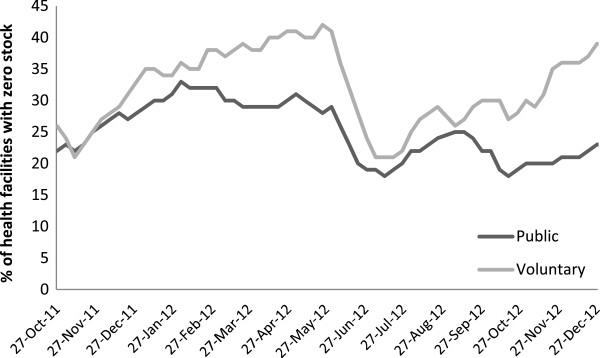
Percentage of health facilities reporting total artemisinin combination therapy stock-out, by health facility type (Public and Voluntary), Tanzania, October 2011 to December 2012.

Weeks where stock increased (compared with the preceding report) were assumed to correspond to deliveries, and a stock-out was identified when the facility reported that they had no stock for one or more colours of blister packs. The study concentrated on total ACT stock-out when all four colours of blister-packs are simultaneously stocked out, since health workers could otherwise cope with a stock-out of only one or a few dose levels by dividing or combining blister packets of other dose levels. Stock-out data by health facility type (public and voluntary) are shown in Figure 1. The 55,047 weeks when a stock-out was reported fell into 11,423 sequences of continuous reporting of stock-out in the same facility. 5,936 of these periods ended with an ACT delivery and the remainder terminated with a censoring event, (either a week where the report was missing, or the end of the study). Kaplan-Meier analysis was used to estimate the distribution of durations of periods of stock-out allowing for these censoring events.

The drug usage rate in facilities that were not stocked-out was estimated as the average rate of decrease in stocks when there was no delivery. This estimated drug usage was added to the reported increase in stocks during weeks when there was a delivery to give an estimate of the total amount of drugs delivered. This study also documented the quantities of ACT shipped and delivered to Tanzania by Novartis Pharma AG, (which at the time of the study was providing all public ACT in Tanzania).

Not all health facilities reported every week in both 2011 and 2012. Table 1 illustrates the percentage of health facilities which failed to provide any report by region for both years. For the complete year of 2012, 11% of health facilities failed to report across the country and 8.6% of health facilities failed to report at all during the entire study period. Wide variations in regional reporting performance were identified with approximately 30% of health facilities in Singida and Dodoma regions failing to report at all, whilst only 0.5% of health facilities in Iringa and Shinyanga regions provided no reports. Short-term average, non-response rate in 2011 showed that 19.8% of weekly SMS request prompts sent received no response, while in 2012 this had increased to 27.7%. However the duration of non-response in any given facility was short (average 1.8 weeks). In instances when there was a missing report, information was interpreted from the stock data of the previous week.

**Table 1:** **Percentage of health facilities never reporting under *SMS for Life***, **by region, 2011 and 2012**

**Region**	**2011 (%)**	**2012 (%)**
Singida	29.6	29.1
Dodoma	29.5	29.8
Morogoro	21.7	21.7
Manyara	14.0	16.0
Arusha	13.2	12.2
Lindi	13.1	10.8
Katavi	11.5	13.5
Mbeya	11.1	12.0
Kigoma	11.0	10.5
Ruvuma	10.1	7.6
Kilimanjaro	9.9	16.0
Dar	9.7	13.7
Tanga	8.9	9.3
Coast	8.1	7.0
Rukwa	8.0	8.0
Mwanza	6.7	8.5
Mtwara	6.5	9.5
Tabora	6.4	8.2
Simiyu	4.4	2.3
Njombe	3.8	4.3
Mara	3.1	4.3
Kagera	2.8	4.0
Geita	1.9	5.5
Iringa	0.6	1.2
Shinyanga	0.5	1.9

Stock-out data by region was derived from the number of weekly SMS received across all health facilities that reported zero stock as a proportion of the total number of SMS received by region across the 15 month study period (see Table 2).

**Table 2:** Regional malaria prevalence and average regional artemisinin combination therapy total stock-out rates in health facilities in 2011-2012

**Mainland region**	**Prevalence**	**% total stock-out**
Tabora	9.2	52.4
Kigoma	26.0	51.6
Ruvuma	12.0	42.6
Geita	31.8	42.5
Shinyanga	6.8	42.1
Mtwara	17.4	41.1
Mwanza	18.6	40.3
Rukwa	4.5	37.8
Simiyu	3.4	36.8
Morogoro	13.0	35.6
Mara	25.4	31.7
Katavi	5.4	29.7
Lindi	26.3	24.7
Tanga	5.6	24.3
Njombe	2.4	22.5
Dar es Salaam	3.6	22.4
Iringa	0.4	21.9
Arusha	0.05	19.7
Kagera	8.3	19.2
Pwani	10.2	18.1
Mbeya	0.05	15.1
Kilimanjaro	0.05	14.8
Manyara	0.9	14.4
Singida	0.2	12.4
Dodoma	2.5	8.7

A simple univariate linear regression analysis was used to study the relationship between malaria prevalence rates and ACT stock-out rates, where regional average ACT stock-outs across the 15 months were regressed against regional malaria prevalence rates for 2011.

## Results

**Figure 2:**
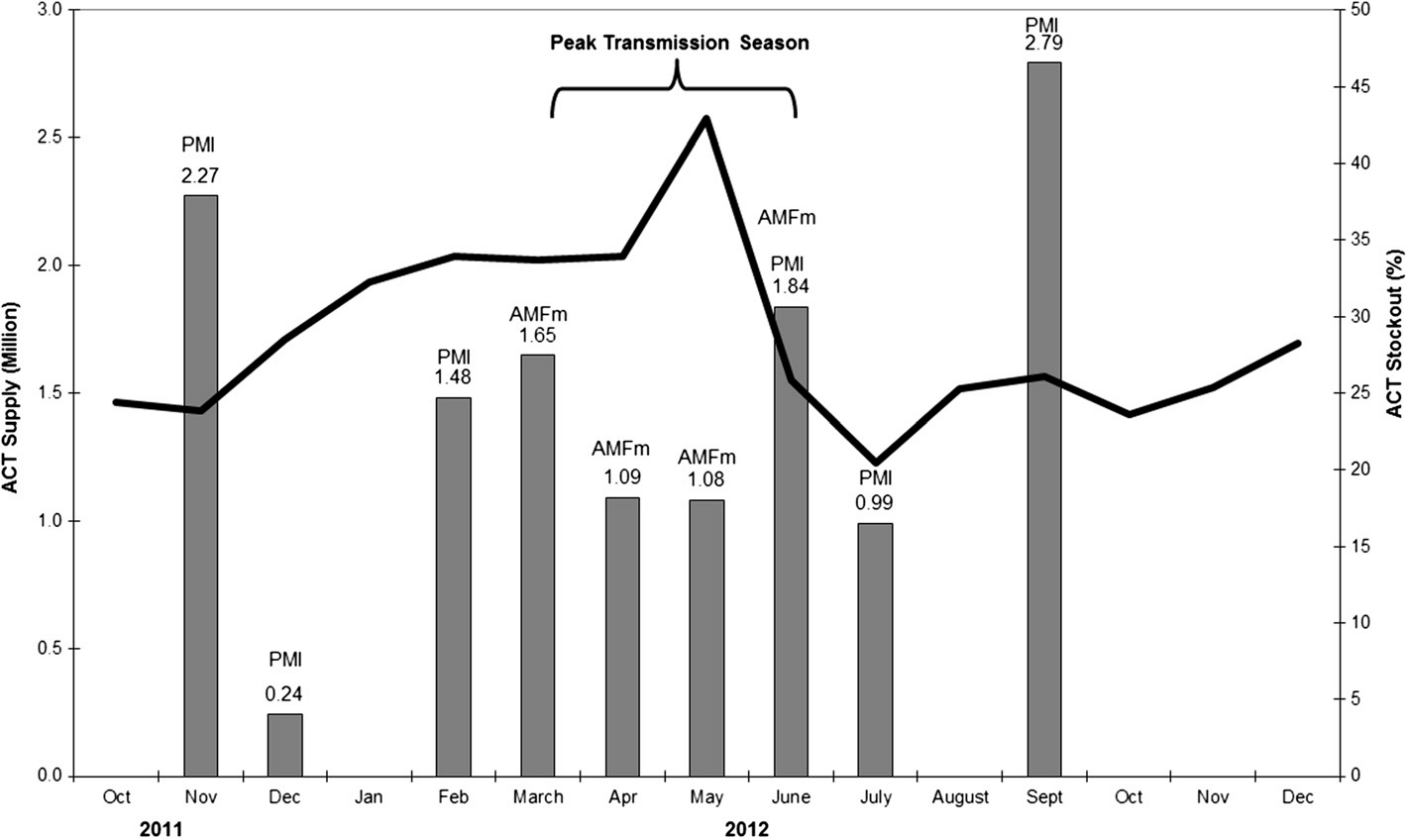
**Percentage of health facilities with total artemisinin combination therapy stock-out, Tanzania, October 2011 to December 2012.** Bars show ACT dose shipments (millions) from the supplier to the public sector (either from PMI or AMFm or both in June 2012).

**Figure 3:**
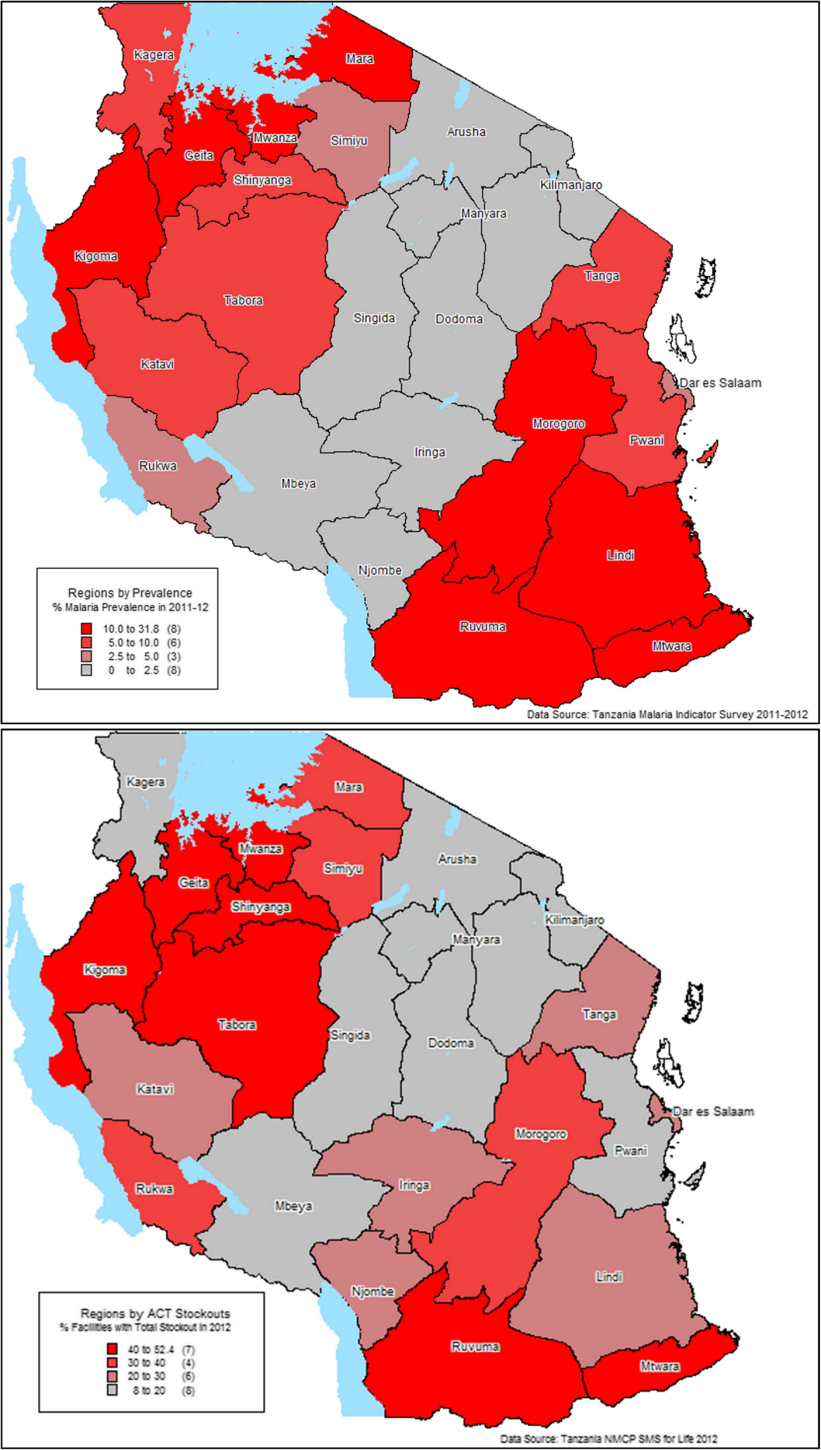
Malaria prevalence (top panel) and average annual total stock-out rate of artemisinin combination therapy in health facilities (bottom panel) by region in 2011–2012 in mainland Tanzania.

During the study period, there were 3030 logins of which 620 logins were by DMOs in districts, 32 by Ministry system administrators and 150 by the NMCP. The proportion of health facilities that recorded simultaneous zero ACT stock of all four doses (total stock-out) as a proportion of all health facility weeks, averaged across the month and across all health facilities by region, is illustrated in Figures 2 and 3.

Figure 2 shows the proportion of health facilities with ACT stock-out (line) while the bars indicate the ACT supply quantities (with corresponding source). For example, in May 2012, 43% of health facilities reported zero ACT stock. For the whole period from October 2011 to December 2012, on average 29% of SMS health facility responses reported a total ACT stock-out. The peak total ACT stock-out period was in the rainy season of April-May 2012, when total ACT stock-out rates reached 40%, implying that nearly half the country was totally stocked out of ACT in the public sector. The estimated median duration of stock-out was six weeks (inter-quartile range 2–14 weeks), with Dar es Salaam experiencing the shortest ACT stock-out and Shinyanga the highest.

Figure 3 compares average stock-out rates from the *SMS for Life* reporting system and malaria prevalence in children under the age of five, as estimated by the 2011–2012 Tanzania National HIV/AIDs and Malaria Indicator Survey across the 25 mainland regions of Tanzania. A simple linear regression analysis suggests that higher malaria prevalence rates correlate positively with higher ACT stock-out rates with every 0.8% increase in prevalence being associated with 1% higher stock-out rates (r^2^ = 0.34, p = 0.002).

The study found that over 90% of ACT doses that were reported as delivered from Novartis in 2012 were documented by *SMS for Life* as passing through the supply chain to the front-line public health facilities. However this is a conservative estimate as it is not adjusted for non-reporting.

## Discussion

The ACT stock-outs in mainland Tanzania have not been well documented in the literature. With the mHealth innovation of *SMS for Life* producing real-time weekly stock reporting, it has been possible for the first time to determine the frequency, magnitude and distribution of stock-outs at the point of delivery in primary care facilities. It was found that across the 15-month study period, average weekly health facility total ACT stock-out rates were 29%, although short-term, non-response among reporting units may mean actual levels are higher. Moreover, of particular concern is that ACT stock-outs averaged around 40% during the rainy season of March-May 2012. These overall results are disappointing considering the pilot programme demonstrated promising results, where stock-outs of three out of the four doses of ACT were greatly reduced or eliminated after the 21-week pilot study period in three districts [22]. A similar pilot study undertaken in Kenya in 87 health facilities across 26 weeks from mid-2011-mid-2012 found that the 5% ACT stock-out identified at the start of the *SMS for Life* programme was completely eliminated by the end [23]. Considering ACT is both lifesaving and available to Tanzania through various donor assistance programmes, these long and persistent stock-outs highlight an unacceptable situation. These are evidence of severe and prolonged health system failures which deserve prompt response at local, national and global level.

**Figure 4:**
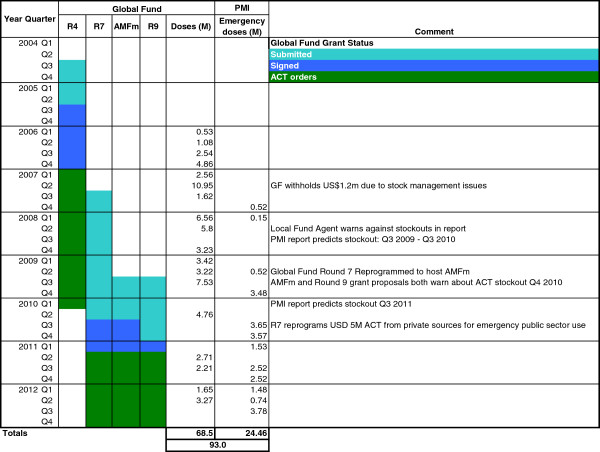
Chronology of important procurement and supply events for artemisinin combination therapy, Tanzania mainland, 2004 to 2012.

Various factors could account for these periods of stock-outs. The current system is designed in such a way that Tanzania forecasts its needs for ACT in discussion with the Global Fund and relies entirely on donor support for its supply [24] (Figure 4). PMI has intervened numerous times in the public sector and in 2012 provided over half the total amount of ACT (55%), whilst the rest was provided under the Affordable Medicines Facility - malaria (AMFm) initiative through the Global Fund [25], aimed at increasing universal access to malaria medicines [26].

Increased reliance on funding partners raises the risk of creating a moral hazard. Records indicate that the Tanzanian government has reduced its malaria budget from USD 5.2 million in 2006–2007 to USD 2.0 million in 2008–2009 [27], a trend identified across other countries that have reduced their domestic funding to health subsequent to increased donor funding [28]. Relying on donor support puts Tanzania at risk of having to abide by external rules and processes; for example, the delay in the Global Fund Round 7 application in 2009 for almost two years was a consequence of changes in grant architecture, including consolidating various rounds that included malaria under one grant as ‘Single Stream Funding’, and having to restructure the grant to host the new AMFm [25,29]. Since the government could not foresee this delay it had no plans to mitigate the shortage. As a result, during 2009 and 2010 Tanzania had to rely on various PMI emergency ACT procurements (another moral hazard), in addition to using residual funds from Round 4 and reprogramming funds from the initial Round 7 grant for emergency ACT procurement (Figure 4). Only in the second quarter of 2011 did AMFm-funded ACT for the public sector enter the market. The entry of AMFm resulted in a dramatic price reduction for ACT in Tanzania. Despite this price reduction in 2012, Tanzania received from AMFm and PMI a monthly average number of 0.9 million doses, 25% less than the estimated requirement of 1.2 million doses per month calculated from the yearly ACT consumption, as estimated in the Round 9 Proposal from Tanzania [4]. The roll out of AMFm into the public sector followed the introduction of AMFm in the private sector. The private sector was less bureaucratically constrained in accessing the AMFm mechanism and was able to move rapidly to acquire co-paid product. This could also explain why the public sector did not receive sufficient quantities in the early stages of AMFm [25].

Another policy change which could impact ACT stock was the rollout of mRDTs where other things being equal, one could expect a reduced ACT demand following an mRDT negative confirmation. Unfortunately *SMS for Life* did not record stocks of mRDTs.

At local level, ACT together with other essential medicines are delivered to health facilities through a “pull” system, requiring health facility workers to manually fill in orders for medicines. They then submit these quarterly to the DMO, who forwards the requests to the MSD to pack individual health kits. The delivery of ACT in this study took place during a change in delivery system; previously the MSD was responsible for delivery from the central warehouse via its nine zonal stores to the districts, where the DMO was then responsible for storing and delivering them to health facilities. However in 2011, the MSD changed policy to ‘Direct Delivery’ where the MSD delivers medicine packages directly to the health facilities [30]. If health facility workers do not place orders, they do not receive medicines or other commodities.

Furthermore, as there is weak reconciliation between the health information system and the medicines ordering system, it is impossible for the health system to assess whether health facility workers are ordering sufficient quantities to cater for the disease profile of the community they serve. The Tanzania procurement and supply logistics system requires that health facility orders be made quarterly, based on the consumption/usage from the previous quarter. The formula for ordering includes a buffer stock of two months, but such ordering systems can be confused by seasonal demand and by stock-outs if they occur. Even with precise and adequate ordering, stock-outs at front-line levels can occur when commodities are not in full supply at regional or national levels. This type of stock-out cannot be managed at the facility level.

Prevalence of malaria and the need for treatment varies geographically across Tanzania. Figure 3 suggests that stock-out rates are higher and of greater duration in areas of high prevalence compared to low prevalence, as might be expected when there are limitations in supply. A key implication of such consistently higher stock-out rates in areas of higher prevalence is that populations, especially children in most need of anti-malarial treatments, are unable to obtain them from their local health facility.

Assuming that Tanzania orders sufficient quantities of ACT, contributing factors to stock-outs could be poor distribution or leakage of ACT. A study of the diversion of anti-malarial medicines in Africa found that the largest share of diverted anti-malarials came from Tanzania, particularly ACT, which were found in private pharmacies in Accra and Lagos, Nigeria [31]. Additionally, there are recent reports that some ACT purchased from a street market in Angola in early 2013 were part of a shipment that was originally donated by either PMI or the Global Fund to Tanzania [32].

However, as this study reported, in 2012 90% of ACT doses shipped were documented by *SMS for Life* as arriving at front-line public health facilities. This figure is surprising considering that 11% of health facilities in 2012 did not report. Several explanations are possible. Firstly, the assumption that any increase in supply from one week to the next corresponds to a delivery maybe incorrect; possibly the increase was just due to undercounting in the previous week. Secondly, additional supplies could have entered the system from previous deliveries in 2011. Lastly, the missing data correspond disproportionately to stock-outs, where some facilities never had ACT and didn't see any point in reporting. Whatever the explanation, the 90% figure is conservative and it is clear that there was plenty of ACT in the system.

All of these factors suggest that the reasons behind stock-outs could include a complex mix of bureaucratic challenges around vertical purchasing and procurement, issues related to distribution strategies in relation to malaria risk, other health system barriers and weaknesses in delivery systems, and illegal diversion. All of these challenges contribute either directly or indirectly to constrain availability of ACT at front-line health facilities.

Transparency of ACT supply is increasingly being demanded as the situation becomes a topic of civil society discussion [33-35]. *SMS for Life* reporting is password-protected and directly accessible to only a limited group of people in the NMCP, the MSD and PSS. However, all involved in forecasting, procurement and delivery of ACT are informed of national stock-outs through the NMCP ACT Technical Working Group, and consequently may be unaware of stock-outs at the dispensary level. Therefore, a policy option available to Tanzania would be to make *SMS for Life*-type data publicly available to enable all stakeholders involved in ACT supply and provision to obtain information on ACT stocks.

To lower the risk of future ACT stock-outs, Tanzania mainland could consider securing subsidized ACT through the AMFm programme and allocate domestic funds to co-finance ACT purchases, thereby creating a national buffer and security stock in addition to buffers built in to the front-line health facility stocks, thereby reducing dependency on donors. However a buffer or security stock is only of value if health system supply barriers can be overcome. *SMS for Life* could also be used for setting more accurate minimum stock levels at the health facility. Most importantly, DMOs and District Health Management Teams (DHMTs) must use the data to make decisions on re-ordering, restocking and redistribution among health facilities to minimize short-term stock-outs between ordering cycles. A pattern of demand could be plotted and modelled to be able to determine trends and seasonal variations in consumption rates, in order to have a more accurate mitigation plan to avoid stock-outs.

This study has some limitations. *SMS for Life* data include short periods of non-reporting by individual health facilities. The high frequency of short-term, non-response in 2012 could have been due to the lack of immediate response to, and follow-up of, non-reporting health facilities by the DMO or the District Malaria Focal Person, or possibly also to a lack of health facility worker administrative data and poor management (e.g., changes in personnel and phone numbers and thus request messages not reaching the right persons). Another possible explanation could be that the regions receiving initial training during the pilot did not benefit from a refresher training and subsequently had higher non-reporting rates; for example, in the initial three pilot regions, Kigoma, Lindi and Morogoro had relatively high short-term, non-response rates of between 26 and 37%. Finally, given that stock-outs in some facilities were prolonged, the health staff may have thought it not worthwhile to report weekly once a stock-out started.

This study was unable to determine whether stock-out rates were higher among non-reporting units compared to those that did report; if that were the case, then the estimate of 30% total ACT stock-out, on average, might be 10-20% too low. Further, this study was not able to examine the stock of ACT in the private sector because such data from *SMS for Life* are not available (however the Independent Evaluation of the AMFm Phase 1 does include information on ACT availability in the private sector [25]). It is likely that ACT availability would be better as the private sector is less affected by constraints in procurement faced by the public sector. In addition, the private sector may have better defined incentives or disincentives structures which encourage efficiency, but this would need to be explored further.

## Conclusions

This study investigated ACT availability across public health facilities in Tanzania for 15 months from October 2011 until the end of 2012 and found that during this period, 29% of health facility-weeks were completely stocked out of the first-line anti-malarial (ACT). An immediate consequence of this was that a significant number of the 14 million cases of malaria that Tanzania experiences that year could not receive first-line treatment at a public health facility. The principle reasons for the failure of the health system to maintain quality of care was due to the heavy reliance on, and difficulties in, compliance with international funding cycles, and fundamental delivery system design flaws at national and subnational level. Both of these risks are addressable, and should be done so urgently by Tanzania and its development partners to avoid a catastrophic resurgence of malaria mortality in the country. With potential ACT resistance, it is imperative that Tanzania secures a stable ACT supply with adequate buffer stock, and implements the use of mRDTs to provide the population with rational use of an affordable treatment against one of the country’s major public health concerns as part of its strategy of ensuring universal coverage for the treatment of malaria. Both ACT and mRDTs need to be monitored weekly at both central and zonal warehouses and at all public front-line health facilities, taking advantage of new mHealth innovations, such as *SMS for Life*. SMS for Life-type systems are useful to understand stock availability at point of delivery. More importantly, such real-time information needs to be directly used for managing the supply chain, not merely to understand by how much it is failing.

## Competing interests

JB and RZ are employees of Novartis Pharma AG, Switzerland.

## Authors’ contributions

DDS, IML, JB, and RZ contributed equally to the conceptualization and design of the approach. IML, TS and DDS analysed the data. IML, WS and DSS wrote the manuscript and all authors reviewed, contributed to and approved the final manuscript.
